# Role of Sex Hormones in the Control of Vegetative and Metabolic Functions of Middle-Aged Women

**DOI:** 10.3389/fphys.2017.00773

**Published:** 2017-10-04

**Authors:** Vincenzo Monda, Monica Salerno, Moscatelli Fiorenzo, Ines Villano, Andrea Viggiano, Francesco Sessa, Antonio I. Triggiani, Giuseppe Cibelli, Anna Valenzano, Gabriella Marsala, Christian Zammit, Maria Ruberto, Giovanni Messina, Marcellino Monda, Vincenzo De Luca, Antonietta Messina

**Affiliations:** ^1^Department of Experimental Medicine, Università degli Studi della Campania “Luigi Vanvitelli”, Naples, Italy; ^2^Department of Clinical and Experimental Medicine University of Foggia, Foggia, Italy; ^3^Department of Medicine and Surgery, University of Salerno, Baronissi, Italy; ^4^Struttura Complessa di Farmacia, Azienda Ospedaliero-Universitaria, Foggia, Italy; ^5^Anatomy Department, Faculty of Medicine and Surgery, University of Malta, Msida, Malta; ^6^Department of Medical-Surgical and Dental Specialties, Università degli Studi della Campania “Luigi Vanvitelli”, Naples, Italy; ^7^Department of Psychiatry, University of Toronto, Toronto, ON, Canada

**Keywords:** autonomic nervous system, hormone-replacement therapy, resting energy expenditure, body composition, assessment of oxidative stress

## Abstract

**Aims:** In women's life, menopause is characterized by significant physiological changes often associated with an increase in body mass and obesity-associated sicknesses. Numerous researches described interdependencies of estrogen deficiency, aging, and resting energy expenditure (REE) downfall in the obesity correlated with the menopause. The aim of this study was to determining whether healthy, obese menopausal women underwent HRT treatment, showed changes in their REE, autonomic asset, and assessment of oxidative stress in comparison with obese pre- and post-menopausal women.

**Methodology:** In this study, we measured the body composition, the REE, the oxidative stress, the diet assimilation, and the autonomic nervous system activity in three groups: pre-menopause women (*n* = 50), post-menopause women following hormone-replacement therapy (HRT; *n* = 50), and post-menopause women not following HRT (*n* = 50).

**Results:** In the group with HRT a significant increase of the sympathetic activity and REE was described. Finally this group showed a notable increment of oxidative stress compared with the others, and utilizing BIA instrument, the free fat mass was increased respect to the fat mass of obese women.

**Conclusion:** The study highlights the importance of the HRT-related physiological changes that influence body weight in menopause women. This results are important because have a practical implications for prevention and/or treatment of the obesity.

## Introduction

In women's life, menopause is characterized by significant physiological changes associated to estrogen deficiency and, subsequently, to the arrest of ovarian activity. In the menopause age, women experienced a general tendency to weight and fat mass (FM) gain (Poehlman et al., 1995). Such increase of adiposity seems to be related to the decline in endogenous estrogen. This hypothesis was tested by several studies using Hormone-Replacement Therapy (HRT). In fact, HRT should prevent or reduce body fat gain, if it is related to decrease of the endogenous estrogen that occurs in menopause time. Unfortunately, there are conflicting data in the literature. Anderson et al. (2001) described that 2-month treatment of HRT in postmenopausal women was not useful for change Body Mass Index (BMI), FM, and Fat-Free Mass (FFM) (O'Sullivan et al., 1995). With a 1 year therapy, Reubinoff et al. (1995) described similar results in body weight increasing and FM values, comparing HRT group with the control group (women without HRT). However, in women not taking HRT a different fat distribution was described with a notable shift from gynoid to android (Wade and Gray, 1979). Another study (Espeland et al., 1997) showed a decrease in body weight in taking HRT women compared to no-taking HRT women along a 3-year period. Differently, another authors described that oral estrogen could lead to the body weight increase, probably reducing lipid oxidation (O'Sullivan et al., 1998). At the light of these findings, in postmenopausal women, it is still unclear the hormone therapy action on body composition.

It seems that body composition may be influenced by ovarian hormones through various possible action routes. In particular, it has been shown that estradiol can inhibit the action of lipoprotein lipase on adipose tissue. This enzyme allow the uptake of fatty acids into adiposity, hydrolyzing running triglycerides (Wade and Gray, 1979). Data from rodent models showed the anorectic effect of estrogen, which decrease voluntary food intake (Dagnault et al., 1993).

Several studies suggested that, in postmenopausal women, weight gain may be due to a rapid Resting Energy Expenditure (REE) decline (Poehlman et al., 1993; Gardner and Poehlman, 1994). As previously described comparing “pre” and “post” menopausal women, the REE decreased by ~420 kcal/day in post-menopause group (Arciero et al., 1993).

Although, the decay in REE is described during the postmenopausal period could be caused by the age, it appears to decline more during the menopause transition (Stern and Murphy, 1972; Bartness and Wade, 1984; Ravussin et al., 1988). During menopause changing, the FM increased thanks to the TREE reduction; besides, they may enhance the occurrence of obesity-related diseases, especially related to worse cardiovascular risk profile (Poehlman et al., 1995) and Type II diabetes (Ravussin et al., 1988). Moreover, the cardiovascular diseases could be increased by the estrogen depletion (Staessen et al., 1989; Vongpatanasin et al., 2001; Weitz et al., 2001). Other studies reported comparable results (Vongpatanasin et al., 2001; Weitz et al., 2001), concluding that the blood pressure in postmenopausal age is reduced by HRT. Furthermore, it has been described that postmenopausal women which did not receive HRT had significantly higher plasma lipids levels (cholesterol and TG) than other women in fertility age, and, more important, than woman which received HRT (El-Sedeek et al., 2010).

Estrogens confer cardioprotection diminishing protein oxidation and antioxidant properties (De Luca et al., 2008; Viggiano et al., 2010). Loss of estrogens may be the cause of oxidative stress occurs at menopause, because of antioxidant effects on low-density lipoproteins (Naruse et al., 2013). Low antioxidant defenses are associated with osteoporosis during the post-menopause period. A study reported that, *in vitro*, oxidative stress modulate the estrogen receptors α and β (Tamir et al., 2002), while antioxidant action of estrogen might partially explain its atheroprotective effect (Clemente et al., 1999), resulting in a decrease of Low Density Lipoprotein (LDL) oxidation (Sack et al., 1994).

Although, the primary action of antioxidant micronutrients in blocking rapid senescent, there are few data concerning the relationship between antioxidant status and oxidative stress in menopausal women. It is clear that there are different factors which contribute to the REE inter-individual variability: sympathetic nervous system (SNS) activity (Francavilla et al., 2007; Monda et al., 2007, 2008b; Messina et al., 2013a, 2014a), FFM (Weyer et al., 1999), and endocrine status (e.g., thyroid hormone).

The SNS is a very important mechanism of control in human body. It has been shown physiological age-related fluctuations which are considered also due to differences in the REE (Day et al., 2005; Monda et al., 2008a; Messina et al., 2012). Heart rate variability (HRV) is a common non-invasive method, useful to provide a comprehensive evaluation of activity of autonomic nervous system (van Ravenswaaij-Arts et al., 1993; Heart rate variability: standards of measurement, physiological interpretation and clinical use. Task Force of the European Society of Cardiology and the North American Society of Pacing and Electrophysiology, 1996; Monda et al., 2006a; Triggiani et al., 2015). Previous studies described that the body fat percentage (Petretta et al., 1995; Triggiani et al., 2017), energy storage (Hirsch et al., 1991), and glucose-induced thermogenesis (Messina et al., 2014b, 2015, 2016) are related to differences in the power spectral components of frequency domain of HRV. A Several investigations, conducting with HRV frequency domain, reported that women, young and obese, show a reduction of sympathetic activity in response of thermogenic variations (i.e., cold or hot exposure; Matsumoto et al., 1999; Francavilla et al., 2008), capsaicin-containing yellow curry diet (Matsumoto et al., 2000), and mixed food intake (Matsumoto et al., 2001).

Although, a relationship between HRV and BMI was described, as written above, other investigations have found no correlation between HRV and BMI (Antelmi et al., 2004; Messina et al., 2013a). Furthermore, Hirsch and Mackintos reported perplexities about the controversial influences of autonomic nervous activity measured by HRV on body weight (Hirsch and MacKintosh, 2003).

This study was aimed at determining whether healthy, obese menopausal women underwent HRT treatment, showed changes in their REE, autonomic asset, and assessment of oxidative stress in comparison with obese pre- and post-menopausal women.

## Methods

### Participants

We enrolled 150 sedentary obese women from the UOC of Dietetic of University of Camapania “L.Vanvitelli” (Italy) that were classified into three groups: 50 pre-menopause women, 50 post-menopause women following HRT, and 50 post-menopause women not following HRT.

The following inclusion criteria were: not smoking, not assuming medication or alimentary additions involved in metabolism or autonomic functions, minerals, or vitamins. The criterium of menopausal status followed the definition of the Harlow et al. (2012). Furthermore, post-menopause women had to follow HRT for at least 2 years or never have followed HRT. The treatment consisted of estrogen and progesterone (estrogen = 0.625 mg/day, progesterone = 2.5 mg/day).

**Figure 1:**
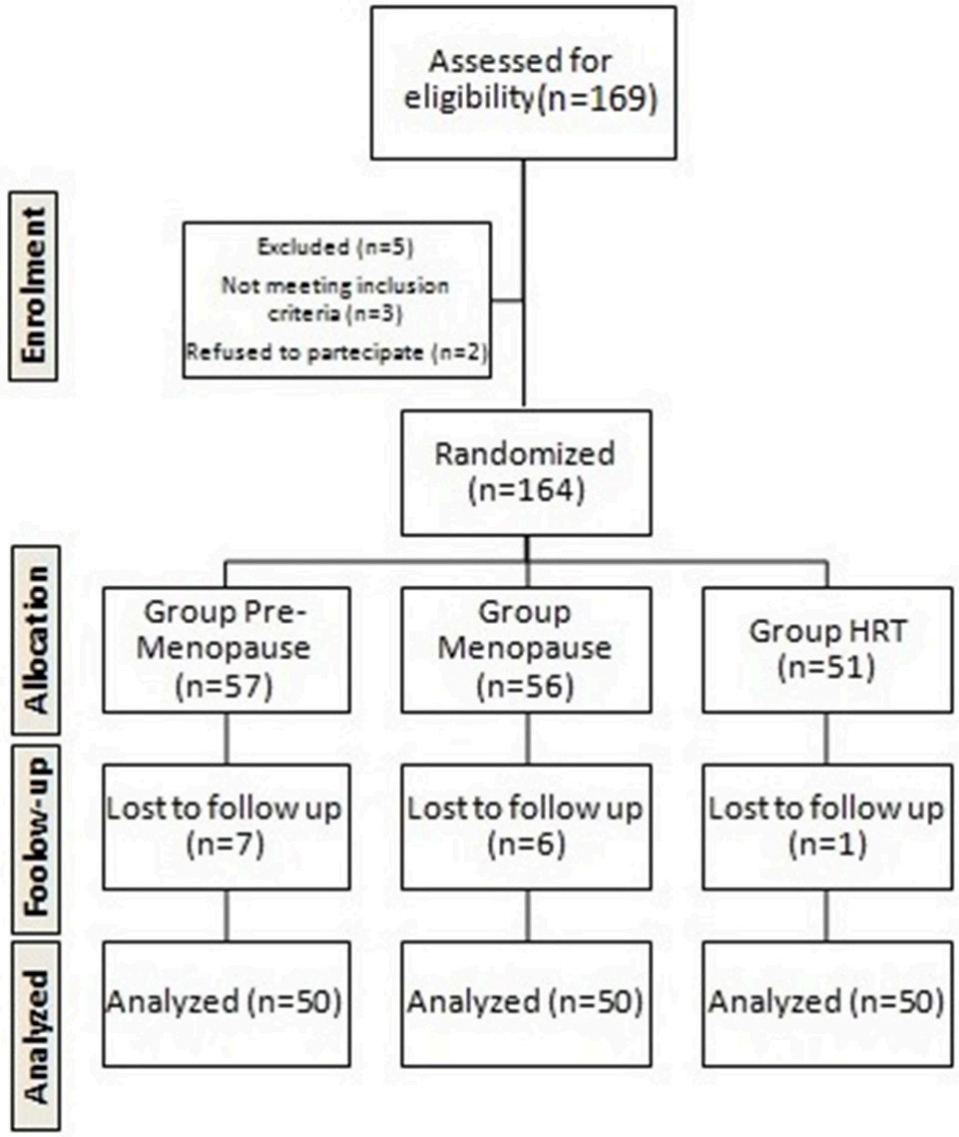
Flow chart of the study.

The randomization was described in the Figure 1 using the Flow Consort diagram.

We provided written and oral information regarding the study protocol, the participants signed the informed consent. At any time, they could leave the study. All the investigation procedures were approved by the local Human Ethical Review Committee and were consistent with the revised declaration of 2013 of Helsinki. Furthermore, all the subjects underwent a clinical assessment to ascertain the absence of any disease. Thus, the subjects enrolled in this study were healthy with a weight constant for a 3 months period.

In Table 1, it was indicated the age and anthropometric values, expressed as means ± SE.

**Table 1:** Age, body mass index (BMI), and blood pressure (systolic, SBP, and diastolic, DBP) in pre-menopause, post-menopause, and HRT women.

**Parameters**	**Pre-menopause (*n* = 40)**	**Menopause (*n* = 40)**	**HRT (*n* = 40)**
Age (years)	43.4 ± 3.9	50.9 ± 3.8	50.6 ± 3.4
BMI (Kg/m2)	30.2 ± 1.46	30.9 ± 0.8	30.8 ± 0.07
SBP (mm Hg)	120.0 ± 10.0	124.0 ± 7.8	124.0 ± 10.0
DBP (mm Hg)	73.0 ± 6.0	72.0 ± 6.4	70.0 ± 9.0

### Indirect calorimetry

We measured REE using a calorimeter (VMax 29, Sensor Medics, USA). It is a computerized system that through a canopy-gas analyzer allowed each measure. Previously each test the correct gas mixture must be establish: thanks to the specific sensors for oxygen and infrared carbon dioxine, the measurement of each analyte was performed. A specific software computed oxygen consumption and carbon dioxide production every minute for 30 min. The first 10 min were discarded, while the mean value of the data during the remaining 20 min was used. REE was expressed as kcal/day and calculated according to Ferrannini (1988). Furthermore, with a kinetic enzymatic method we estimated the urea values in urine previously collected in a 12-h interval (from 8:00 to 20:00) (Urea SYS 1, Boehringer Mannheim, Mannheim, Germany). REE was linearly adjusted for fat-free mass and age, according to Ravussin (Ravussin et al., 1988). Adjusted REE was the mean REE plus individual REE value, measured for each subject after a 12 h period of overnight fasting. All the measurements were made between 8:00 and 11:00 a.m.

### HRV

We evaluated HRV by a 5-min electrocardiogram (ECG, delta-1 plus, Cardioline, Milan, Italy); for data acquisition and analysis, a convention tool made with LabView (National Instruments, Texas, USA) was used. During the tests the R waves were automatically recorded. The R-R range was measured, interpolated, and resampled to obtain a constant-time-based signal (100 ms). The Fast Fourier Transform (FFT) algorithm was applied to this signal to obtain the Power Spectral Density (PSD). The PSD was divided into bands: in particular, we were interested to the low-frequency power (LF; 0.04–0.15 Hz) and the high-frequency power (HF; 0.15–0.40 Hz). The LF, HF, and the LF/HF ratio were used to estimate the sympathetic and parasympathetic activities. All the HRV tests were performed following the guidelines of the Task Force of the European Society of Cardiology and the North American Society of Pacing and Electrophysiology (Heart rate variability: standards of measurement, physiological interpretation and clinical use. Task Force of the European Society of Cardiology and the North American Society of Pacing and Electrophysiology, 1996).

### Body composition

We used conventional Body Impedance Analysis (BIA) to assess body composition (BIA 101 RJL, Akern Bioresearch, Firenze, Italy), under manufacturer's instructions. For these tests, the experimental procedures are previously described (Savastano et al., 2010). Patients did not perform an intense physical activity, maintaining an usual consumption of caffeine 3 days before the experiment. All tests were performed with empty bladder and after an overnight fasting. From bioelectrical measurements and anthropometric data, the software provided by the manufacturer computed the body composition using validated predictive equations for fat free mass (FFM), total, fat mass, and total body water. All the participants underwent the bioimpedentiometry between the eighth and eleventh day from the onset of the menstrual cycle. We assure that subjects were in an optimal state of hydration for BIA (Matsumoto et al., 1999), asking them to be fasting for 12 h, to not assume drinks for 4 h and to not assume contraceptives over the last 3 months. For sake of brevity, we only reported the percentage of FFM (calculated as FFM divided by total body weight).

### Free radical analytical system 4 (Fras-4)

We measured total Reactive Oxygen Species (ROS) production by Free Radical Analytical System 4 (d-ROMs test kit; Diacron, Grosseto, Italy). The total ROS production was measured whith d-ROMs test kit on blood samples. The reaction of ROS and ROS derivatives with a suitably buffered chromagen yields to a colored compound photometrically measured at his maximum absorbency peak (505 nm). According to the Lambert-Beer law, this value is directly proportional to ROS concentration. ROS production was expressed as Carr Units, as established by the manufacturer.

### Other parameters

We measured blood pressure by the Riva–Rocci method: subjects had to stay seated for 5-min at rest, before the measurement using a standard mercury sphygmomanometer. We computed the average of two different measurements as representative of the patient's blood pressure (Table 1). Blood tests showed normal values for cholesterol, thyroid hormones, triglycerides, and azotemia.

### Statistical analysis

We analyzed data using IBM SPSS Statistics for Windows (Version 20.0. Armonk, NY: IBM Corp). Analysis of variance (ANOVA) tested the differences among dependent variables for the three groups (pre-menopause, post-menopause, and HRT). When significant, a Bonferroni multiple comparisons *post-hoc* test identified significant differences between groups. Multivariate regression analysis was performed in order to evaluate the role of confounding factors on results. All data were reported as means ± standard error. Statistical significance was considered for *p* ≤ 0.05.

## Results

**Figure 2:**
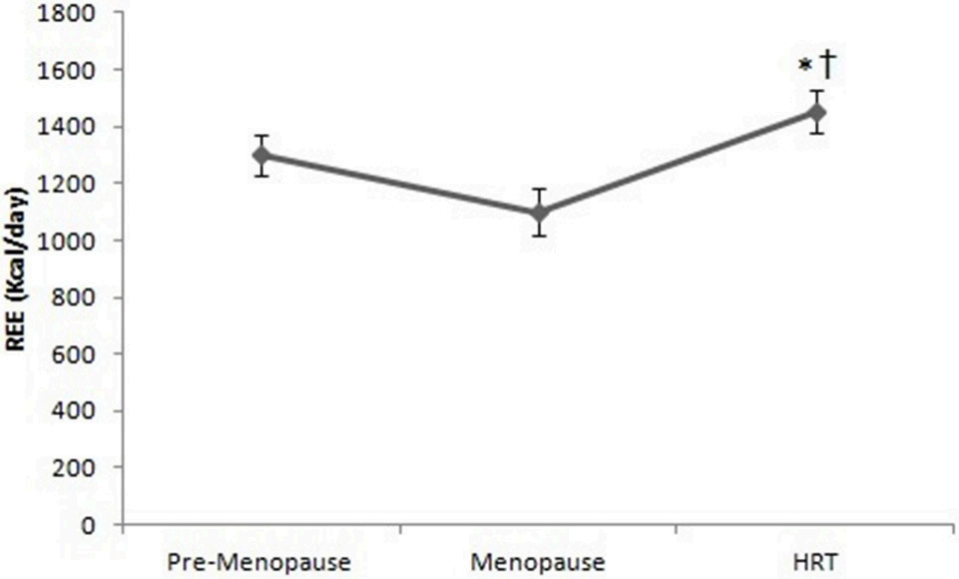
Resting energy expenditure in pre-menopause, post-menopause, and HRT women. ^*^HRT vs. pre-menopause (*p* < 0.01). ^†^HRT vs. post-menopause (*p* < 0.01).

As shown in Figure 2, the REE of HRT women is higher than of pre-menopause, post-menopause women. Analysis of variance (ANOVA) showed a significant effect [*F*_(2, 147)_ = 16.9, *p* < 0.01]. *Post-hoc* test showed a difference between HRT and pre-menopause and between HRT and post-menopause women.

**Figure 3:**
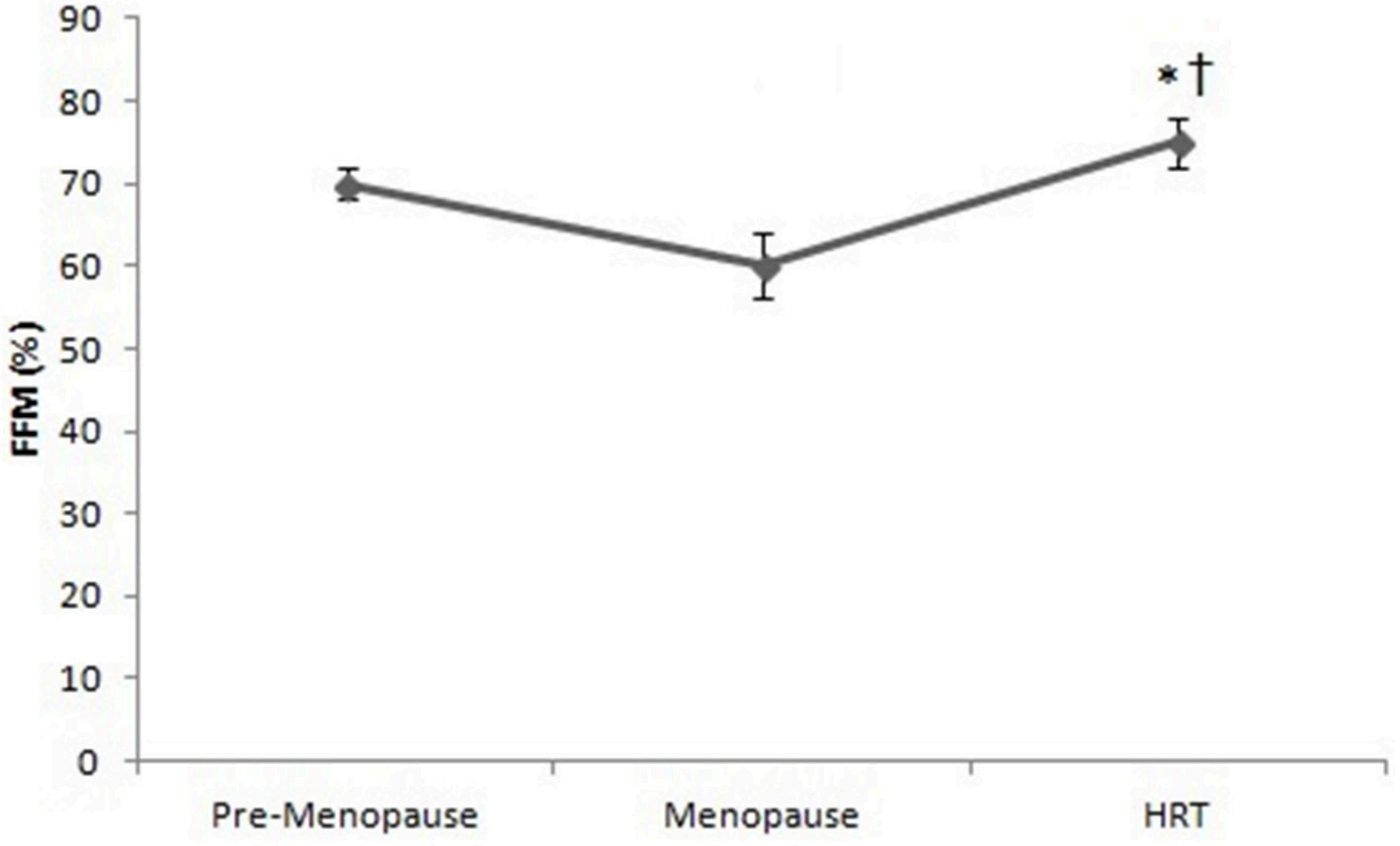
Free Fat Mass (FFM) in pre-menopause, post-menopause, and HRT women. ^*^HRT vs. pre-menopause (*p* < 0.01). ^†^HRT vs. post-menopause (*p* < 0.01).

Figure 3 shows that the percentage of FFM of HRT is higher than of pre-menopause and post-menopause women. ANOVA showed significant effect [*F*_(2, 147)_ = 3.22, *p* < 0.01]. *Post-hoc* test showed a difference between HRT and pre-menopause and between HRT and post-menopause women.

**Figure 4:**
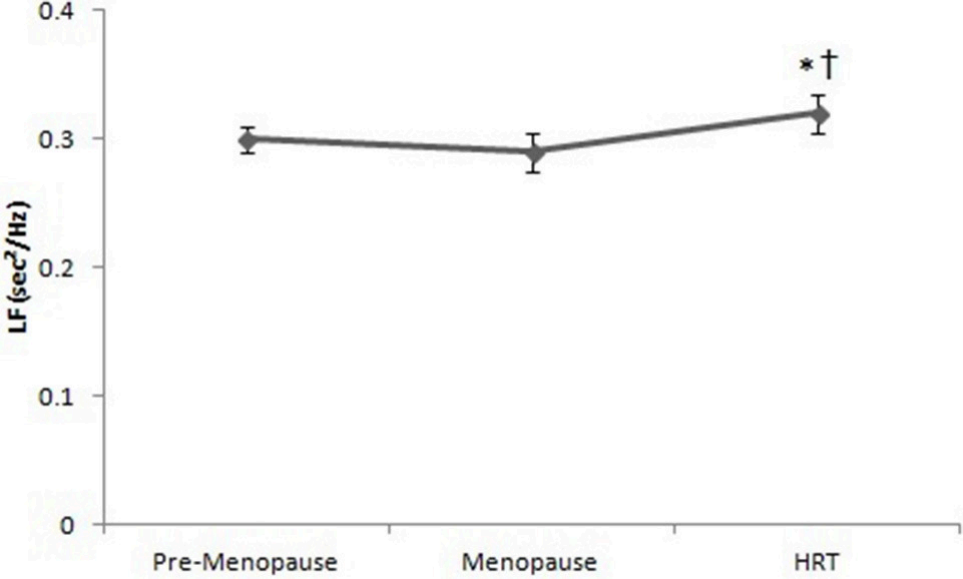
Low frequencies of heart rate variability in pre-menopause, post-menopause, and HRT women. ^*^HRT vs. pre-menopause (*p* < 0.01). ^†^HRT vs. post-menopause (*p* < 0.01).

Figure 4 reports the values of LF in HRT are higher than of pre-menopause and post-menopause women. ANOVA showed significant effect [*F*_(2, 147)_ = 7.44, *p* < 0.01]. *Post-hoc* test showed a difference between HRT and pre-menopause and between HRT and post-menopause women.

**Figure 5:**
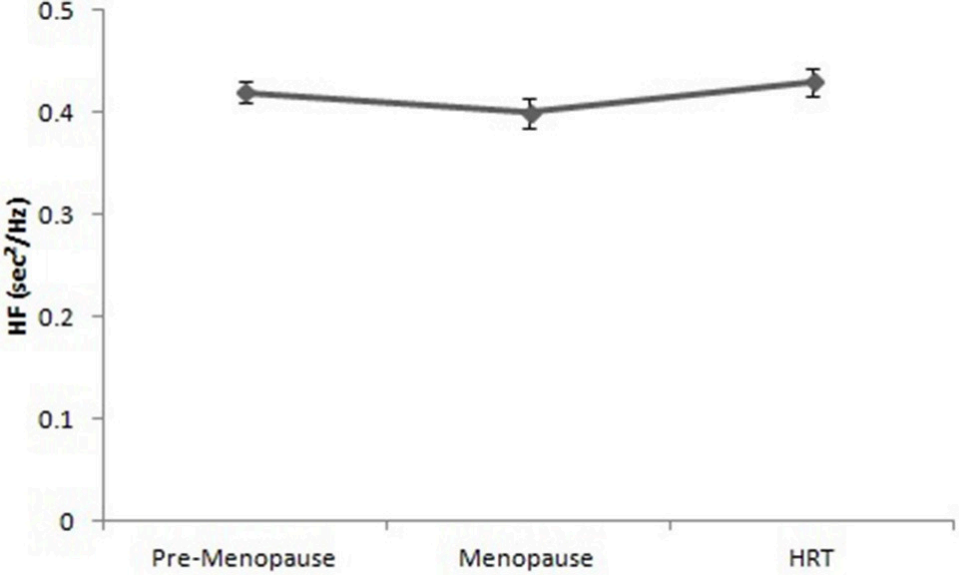
High frequencies of heart rate variability in pre-menopause, post-menopause, and HRT women.

Figure 5 reports the values of HF of pre-menopause, post-menopause, and HRT women. ANOVA showed no significant effect [*F*_(2, 147)_ = 12.23, *p* = 0.16].

**Figure 6:**
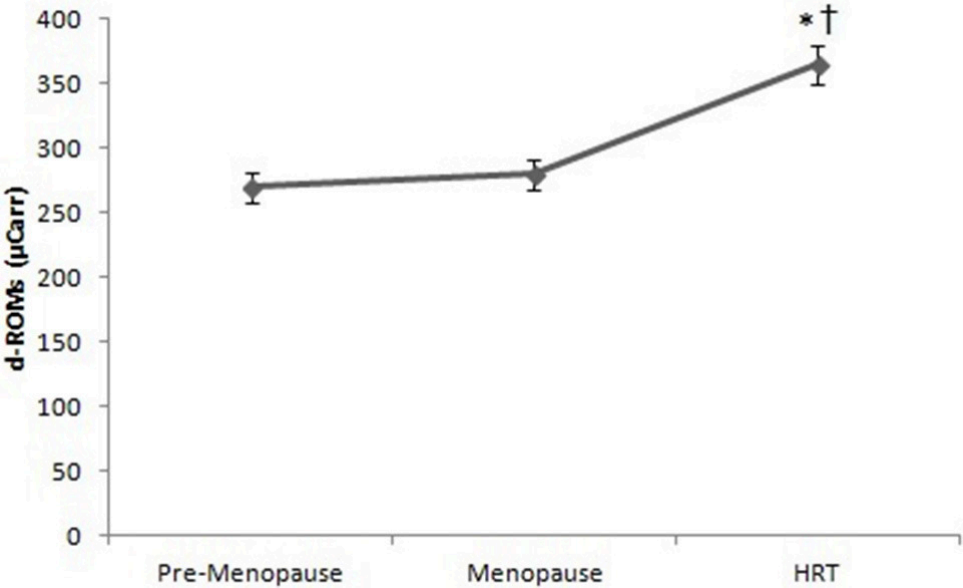
d-ROMs test in pre-menopause, post-menopause, and HRT women. ^*^HRT vs. pre-menopause (*p* < 0.01). ^†^HRT vs. post-menopause (*p* < 0.01).

Figure 6 reports the values of d-ROMs test in HRT are higher than of pre-menopause and post-menopause women. ANOVA showed significant effect [*F*_(2, 147)_ = 2.25, *p* < 0.01]. *Post-hoc* test showed a difference between HRT and pre-menopause, post-menopause women.

## Discussion

This study reports for the first time the difference in REE, sympathetic function, and oxidative stress between women in pre-menopause, menopause or under HRT. Thanks to this experimentation, HRT women have a variation in the vegetative modulation. Moreover, the same group shows an increment of autonomic control respecting the sympathetic component. The increase of the sympathetic branch is an important factor in maintaining the highest REE in women in HRT compared to premenopausal and menopausal women. These experimental data demonstrate that in the premenopausal and menopausal groups the autonomic activity and the vegetative control are lower than the HRT group.

The modification of metabolically active components lead to the age-related decline in REE; these metabolic alterations can be induced by menopause. For instance, the arrest of sex hormones to postmenopausal levels in young healthy women reduces REE, theoretically because of a reduction of sympathetic activity (Day et al., 2005; Monda et al., 2006a,b; Messina et al., 2013b; Viggiano et al., 2014).

In these experimental conditions, even if the age lead to modification the sex hormones level, we confirmed the lack of REE- and FFM-decline in obese women. Probably, the explanation is that the sympathetic nervous system, involved in the control of body weight, does not diminish during the aging (Tentolouris et al., 2006). This high sympathetic activity could also clarify the absence of FFM-decline, because the trophism of skeletal muscle (a fundamental component of FFM) is surely involved by the sympathetic discharge (Ciccone et al., 2013).

The originality of the present experimentation is to highlight the difference sympathetic activity induced by HRT and then on the relationship between the sympathetic nervous system and REE. As previously described, the sympathetic activity has a notable impact on eating behavior, for example increasing the thermogenesis (Bray, 2000).

These results are in accord with the theory that a diminution in autonomic activity could play an important role in the increase in food intake and in the induction weight gain in menopausal women (Viggiano et al., 2009).

This experimentation, on the one hand, underlines that the activity of autonomic nervous system is in relationship with the body weight in HRT and menopause. In this manner, it is recognized his role in obesity or/and in aging.

On the other hand, the role of estrogen as an antioxidant *in vivo* is a matter of debate (Santanam et al., 1998; Di Bernardo et al., 2014; Esposito et al., 2014; Monda et al., 2014). The beneficial protecting effect of HRT is still not clear. Several studies described the beneficial effect for the prevention of coronary artery disease, morbidity, and mortality (Grady et al., 1992). To date, the preventive role of HRT for cardiovascular events is not support by literature data from clinical trials (Hemminki and McPherson, 1997). Furthermore, there is only one study with randomized data that does not support the useful effect in postmenopausal women with coronary heart disease (Hulley et al., 1998).

As previously described the assumption of estrogen is a risk factor for breast cancer because it increases the cell proliferation and the random errors during DNA replication (Henderson and Feigelson, 2000). However, estrogen metabolism also generates reactive oxidative species (Yager and Liehr, 1996; Ambrosone, 2000). The levels of reactive oxidative species are very important for cell signaling processes: while a modest are necessary for cell life (Martin and Barrett, 2002), excess can damage DNA, lipids, and proteins (Loft and Poulsen, 1996). In this contest, the assumption of estrogens generates reactive quinones capable of forming adducts with DNA and of participating in redox cycling (Yager, 2000; Li Volti et al., 2011). In consequence, similarly to the signaling processes involved in cell growth or apoptosis, the estrogen metabolites play an important role, directly or indirectly, in oxidative damage to cellular components (Martin and Barrett, 2002; Salomone et al., 2013).

As previously described, the equine estrogens are often contained in the HRT preparations: comparing the metabolites of equine estrogens with the human, a greater potential for causing oxidative damage was described (Zhang et al., 1999; Tibullo et al., 2013; Pomara et al., 2016). A metabolite of equine estrogen, 4-hydroxyequilenin, has been shown to cause oxidative damage and single-strand breaks in λ phage DNA (Chen et al., 1998) and in breast cancer cell lines, especially ER-positive cell lines (Liu et al., 2002).

## Conclusion

In conclusion, the effect of HRT remains controversial. In a particularly moment of women life such as postmenopausal, the optimal assumption of antioxidant micronutrient could represent an important tool to fight the oxidative stress produced by hormonal modifications.

As previously described in metabolic studies, the soy, thanks to isoflavones, has an important lipid-lowering effect, favors vasodilatation, and arterial compliance and contributes regulating fasting glucose and insulin levels in humans. In addition, phytoestrogens by their estrogenic properties may favorably affect muscle mass. However, it is not clear if isoflavone supplementation could increase FFM. Estrogens are steroid hormones, which mediate their action by binding to a number of tissue receptors, including specific nuclear estrogen receptors (ER; α and β) and plasma membrane-associated ER. ER α and β function as transcription factors once bound to their ligand. ER α and β are expressed and localized within skeletal muscle tissue and in tendons and ligaments, suggesting a direct effect of estrogen (Aubertin-Leheudre et al., 2007). In this sense, as previously described, the soy protein supplementation has an effect on hip lean mass in perimenopausal women 40 g/day for 24 weeks; and on lean body mass in elite athletes, 1.5 g/kg/day for 8 weeks. However, the findings of the present study agree with other literature data. In fact, it has been well documented that menopause is associated with a decrease in REE (Aubertin-Leheudre et al., 2007).

This type of research analyzed aging effects on fertile women and on menopause women: in accord with previous research (Monda et al., 2006a, 2008a), aging modifies the oxygen waste adaptation and the activity of autonomic nervous system.

Under these conditions, at the light of these findings, the nutrition represents an innovative and alternative way in preventing aging. Finally, these experimental data are important because have a practical implications for prevention and/or treatment of the obesity: a pandemic disease widespread in recent years, not only in the Western World, but in developing countries as well.

## Author contributions

VM, IV, AnVi carried out biological assays and, with the contribution of AM, MR carried out the patient evaluations. FS, MF, GC, AnVi, GM participated in the design of the study. AT and CZ performed the statistical analysis. GiMe, MM, VDL, AM, VM conceived of the study, participated in its design, and coordination, and helped to draft the final manuscript. All authors read and approved the final manuscript.

### Conflict of interest statement

The authors declare that the research was conducted in the absence of any commercial or financial relationships that could be construed as a potential conflict of interest.
